# Hydrostatic pressure induces osteogenic differentiation of adipose-derived mesenchymal stem cells through increasing lncRNA-PAGBC

**DOI:** 10.18632/aging.103448

**Published:** 2020-07-13

**Authors:** Jiangying Ru, Lieping Guo, Yinjun Ji, Yunfei Niu

**Affiliations:** 1 Department of Orthopedics, The Affiliated Hospital of Yangzhou University, Yangzhou University, Yangzhou 225001, China; 2 Department of Oncology, Shanghai Eastern Hepatobiliary Surgery Hospital, Second Military Medical University, Shanghai 200433, China; 3 Department of Trauma Orthopedics, Changhai Hospital, Second Military Medical University, Shanghai 200433, China

**Keywords:** hydrostatic pressure (HP), adipose-derived mesenchymal stem cells (AMSCs), PAGBC, RUNX2, miR-133b

## Abstract

Induced osteogenesis of adipose-derived mesenchymal stem cells (AMSCs) has been used to facilitate bone regeneration. Specifically, hydrostatic pressure (HP) has been implicated as a key regulator of AMSC differentiation, whereas the mechanisms that underlie the effects of HP on osteogenesis of AMSCs are not fully understood. Long noncoding RNAs (lncRNAs) are emerging regulators for osteogenic differentiation from AMSCs. In the current study, we found that lncRNA-PAGBC was a specific lncRNA that significantly upregulated during osteogenic differentiation of AMSCs based on published database. HP increased lncRNA-PAGBC, which is a competitive endogenous RNA (ceRNA) that binds to the osteogenesis-inhibitory microRNA, miR-133b, to regulate osteogenic differentiation of AMSCs. Moreover, a key osteogenesis-trigger gene, runt-related transcription factor 2 (RUNX2), was identified as a target gene for miR-133b. Suppression of RUNX2 by miR-133b caused impaired osteogenic differentiation of AMSCs. Furthermore, lncRNA-PAGBC overexpression upregulated, whereas lncRNA-PAGBC silencing decreased the expression of RUNX2 through miR-133b. Together, these data suggest that HP induces osteogenic differentiation of AMSCs through increasing lncRNA-PAGBC.

## INTRODUCTION

Cell-based tissue-engineering techniques are attractive methods for improving tissue regeneration in patients [1]. Mesenchymal stem cells (MSCs) are the most commonly studied and applied cells in boosting tissue regeneration [2]. Specifically, adipose-derived mesenchymal stem cells (AMSCs) have the advantage of being abundant, accessible and functional [3–9].

Tissue engineering strategies have been widely used to facilitate natural bone regeneration processes to fill bone defects resulting from trauma, resection of tumor and severe infection [10]. Besides osteogenic cells, osteoconductive scaffolds and osteogenic cytokines, mechanics are also critical factors for optimal and constitutive tissue engineering [11]. By Wolff's law, the geometrical remodeling of bone responds faithfully to mechanical loads in a dynamic manner [10]. Moreover, the mechano-transduction theories have been recently developed to describe how physical forces are converted into biological signals to trigger cellular responses [12]. Hydrostatic pressure (HP) is a constant strain on bone cells inside the body. HP constitutes a quarter of the systemic blood pressure for regulating the dynamic homeostasis of bone [13]. Nevertheless, the exact way by which HP affects osteogenic differentiation of MSCs is not fully understood.

MicroRNAs (miRNAs) are 20~22 nucleotides long non-coding RNAs [14]. Most of miRNAs combine to 3'-untranslated region (UTR) of genes by imprecise binding, resulting in silence of the genes by alternation of spatial structure [14]. MiRNAs play an important role in various biological processes, such as regulation of cell differentiation, determination of cell identity, modulation of apoptotic cell death, cell migration and cell cycles, et al [15–18]. Specifically, miR-133b is a miRNA that suppresses osteogenic differentiation [19]. Moreover, a key osteogenesis-trigger gene, runt-related transcription factor 2 (RUNX2), was found to be the target for miR-133b during osteogenesis [19]. Similarly, miR-133 was found to affect fracture healing through RUNX2 in another study [20].

Long noncoding RNAs (lncRNAs) are non-protein coding RNAs of more than 200 nucleotides in length [21], and are emerging regulators for osteogenic differentiation from AMSCs [22–24]. PAGBC was a specific lncRNA that significantly upregulated during osteogenic differentiation of AMSCs, based on published database [25]. However, the involved mechanisms have not been studied.

Here, we showed that HP increased lncRNA-PAGBC, which is a competitive endogenous RNA (ceRNA) that binds to the osteogenesis-inhibitory microRNA, miR-133b, to regulate osteogenic differentiation of AMSCs. Moreover, suppression of RUNX2 by miR-133b caused impaired osteogenic differentiation of AMSCs. Furthermore, lncRNA-PAGBC overexpression upregulated, whereas lncRNA-PAGBC silencing decreased the expression of RUNX2 through miR-133b.

## RESULTS

### HP upregulates PAGBC and induces osteogenic differentiation of AMSCs

**Figure 1:**
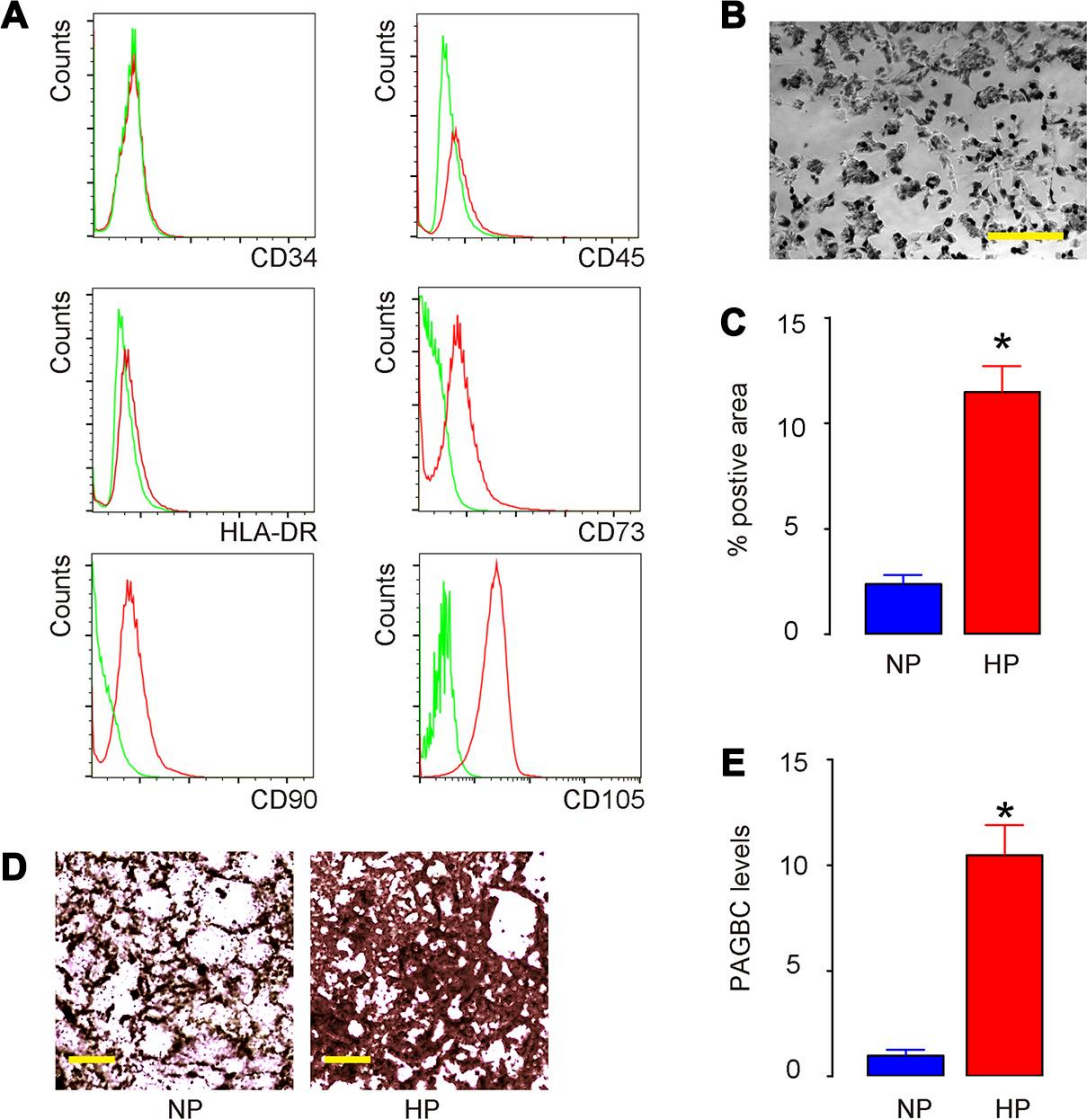
**HP upregulates PAGBC and induces osteogenic differentiation of AMSCs.** (**A**) Isolated human AMSCs were validated for MSC property (positive for CD73, CD90 and CD105, negative for CD34, CD45 and HLA-DR) by flow cytometry. (**B**) AMSCs in culture. (**C**–**E**) AMSCs were cultured in osteogenic differentiation media under normal pressure (NP) culture versus HP culture. Osteogenic differentiation of AMSCs was determined by Von kossa staining, shown by quantification (**C**) and by representative images (**D**). (**E**) RT-qPCR for PAGBC. N=5. *p<0.05. Scale bars are 50μm.

AMSCs were isolated from healthy donor and one clone was selected, after validation for MSC property (positive for CD73, CD90 and CD105, negative for CD34, CD45 and HLA-DR) by flow cytometry (Figure 1A). Next, AMSCs were cultured in osteogenic differentiation media under normal pressure (NP) versus HP (Figure 1B). We found that HP significantly increased osteogenic differentiation of AMSCs by Von kossa staining, shown by quantification (Figure 1C) and by representative images (Figure 1D). Next, in order to find the HP-regulated lncRNAs associated with osteogenesis, we obtained candidate lncRNAs that significantly upregulated during osteogenic differentiation of AMSCs from published database [25]. In these candidates, PAGBC was found to be significantly upregulated by HP (Figure 1E). Thus, HP upregulates PAGBC and induces osteogenic differentiation of AMSCs.

### PAGBC is a ceRNA for miR-133b in AMSCs

**Figure 2:**
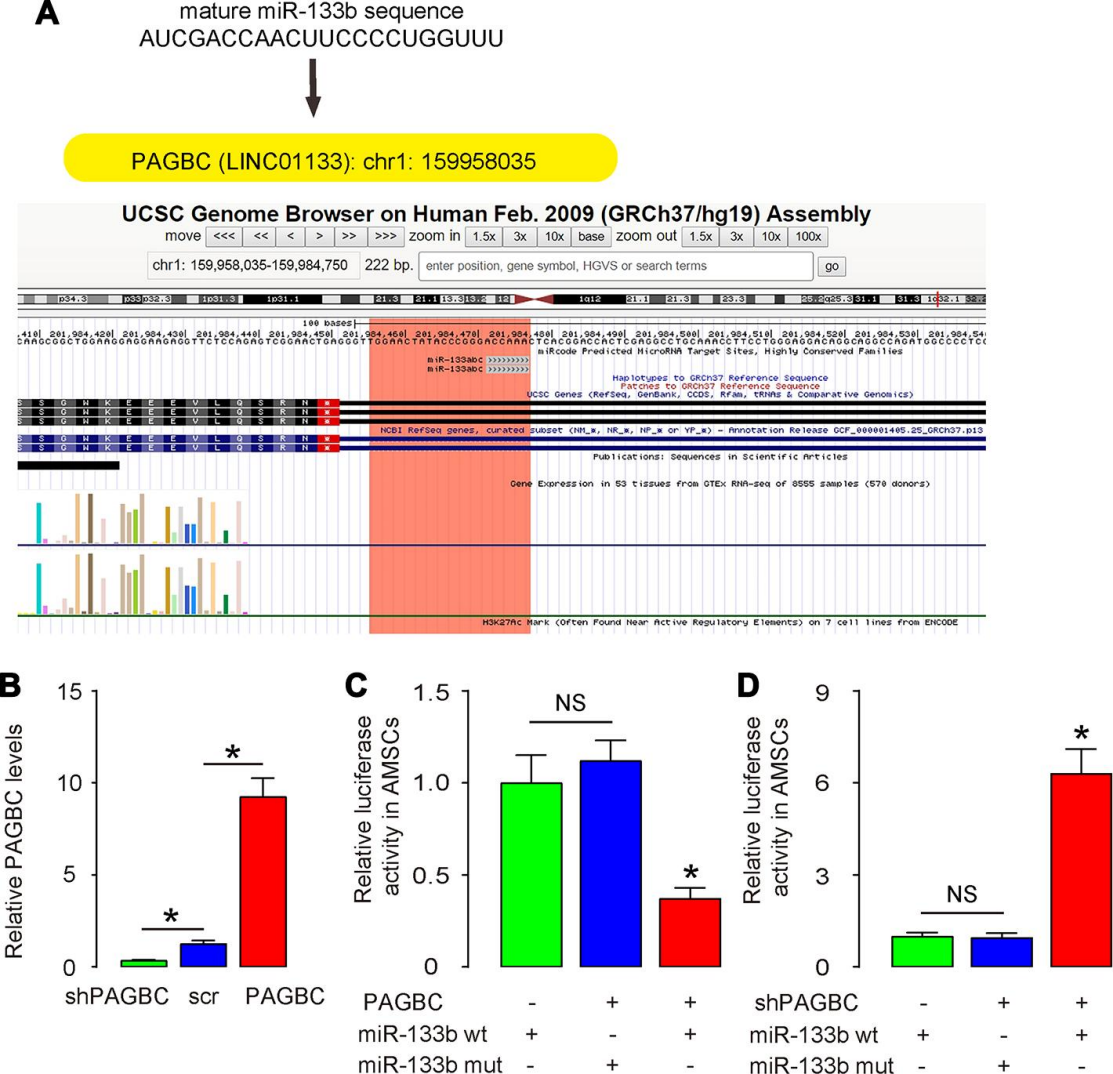
**PAGBC is a ceRNA for miR-133b in AMSCs.** (**A**) MiR-133b was a specific PAGBC-targeting miRNA, analyzed by miRcode. (**B**) Plasmids that overexpress PAGBC or deplete PAGBC were prepared. Plasmids carrying a scramble sequence were used as controls. These plasmids were used to transfect AMSCs and RT-qPCR for PAGBC was assessed in these cells. (**C**, **D**) We prepared luciferase reporter for wildtype (wt) miR-133b and miR-133b with a mutant at the PAGBC binding site (mut). The luciferase reporter assay was performed on AMSCs, using either miR-133b wt (**C**) or miR-133b mut (**D**). N=5. *p<0.05. NS: non-significant.

Using miRcode (http://www.mircode.org/mircode), we found that most of the targeting genes of PAGBC were not associated with osteogenic differentiation. However, miR-133b was a specific PAGBC-targeting miRNA (Figure 2A), which suppressed osteogenic differentiation through RUNX2, a key osteogenesis-trigger gene [19]. Hence, we hypothesized that PAGBC may compete with RUNX2 for miR-133b binding to increase free RUNX2 to promote osteogenic differentiation of AMSCs. To prove it, first we examined whether PAGBC may be a ceRNA for miR-133b in MSCs. We prepared plasmids that overexpress PAGBC or deplete PAGBC. Plasmids carrying a scramble sequence were used as controls. The potential of these plasmids to alter PAGBC levels correspondingly was validated by RT-qPCR (Figure 2B). Next, we prepared luciferase reporter for wildtype (wt) miR-133b and miR-133b with a mutant at the PAGBC binding site (mut). The luciferase reporter assay was performed on AMSCs, showing that transfection of PAGBC markedly reduced the luciferase activity of miR-133b-wt (p<0.05), whereas the luciferase activity of the miR-133b-mut was unaffected (Figure 2C). Moreover, transfection of shPAGBC markedly increased the luciferase activity of miR-133b-wt (p<0.05), whereas the luciferase activity of the miR-133b-mut was unaffected (Figure 2D). Together, these data suggest that PAGBC is a ceRNA for miR-133b in AMSCs.

### HP induces osteogenic differentiation of AMSCs through PAGBC

**Figure 3:**
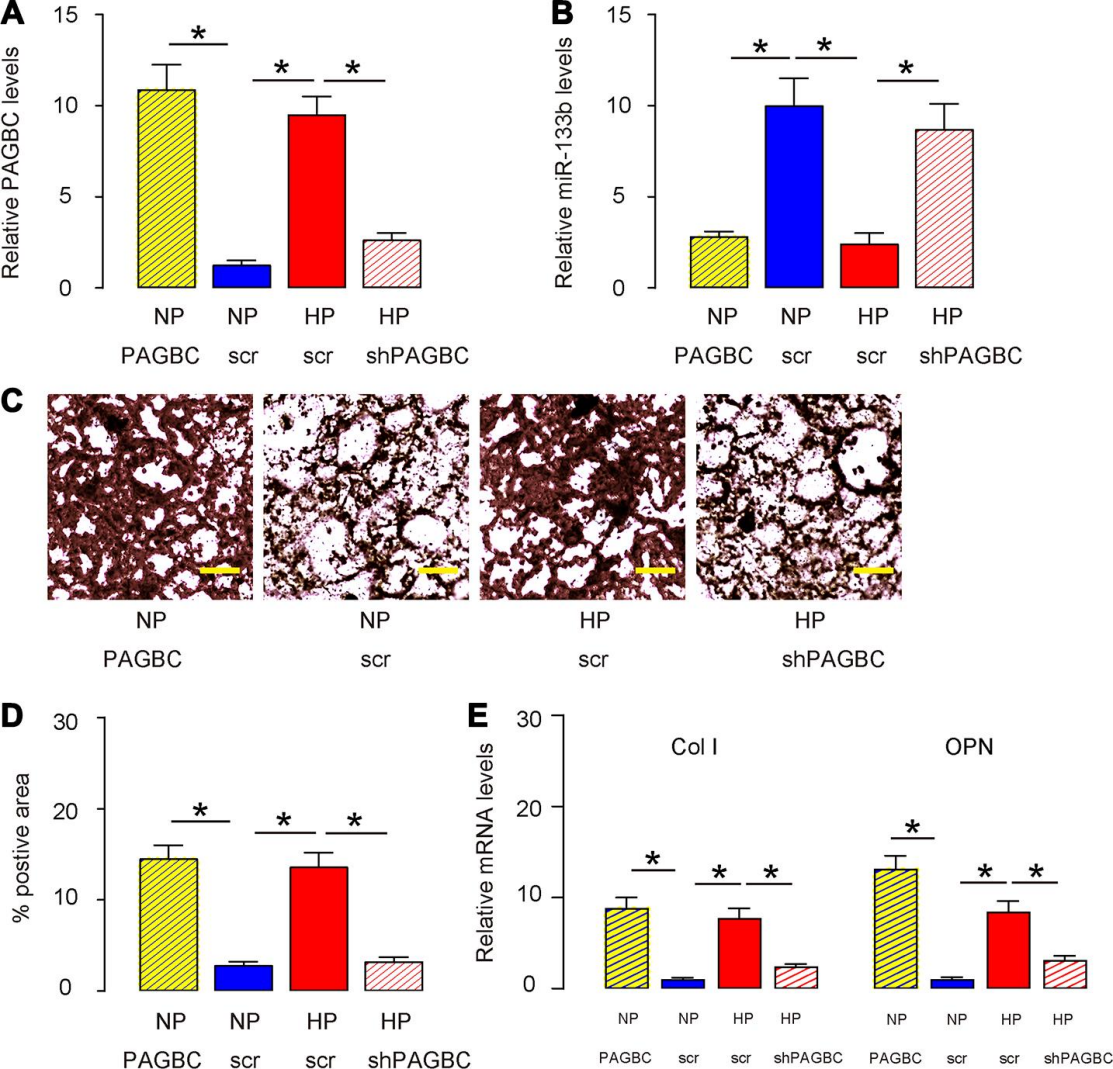
**HP induces osteogenic differentiation of AMSCs through PAGBC.** AMSCs transfected with scr were kept in either NP or HP, while AMSCs transfected with PAGBC were kept in NP, AMSCs transfected with shPAGBC were kept in HP, to compare with AMSCs in NP and AMSCs in HP, respectively. (**A**, **B**) RT-qPCR for PAGBC (**A**) and miR-133b (**B**) in AMSCs. (**C**, **D**) Osteogenic differentiation of AMSCs was determined by Von kossa staining, shown by quantification (C) and by representative images (**D**). (**E**) RT-qPCR for osteogenesis-associated genes, collagen I (Col I) and osteopontin (OPN). N=5. *p<0.05. Scale bars are 50μm.

Next, we assessed whether HP may induce osteogenic differentiation of AMSCs through PAGBC. AMSCs transfected with scr were kept in either NP or HP, while AMSCs transfected with PAGBC were kept in NP, AMSCs transfected with shPAGBC were kept in HP, to compare with AMSCs in NP and AMSCs in HP, respectively. HP induced significantly increases in PAGBC in AMSCs, which were significantly attenuated by shPAGBC (Figure 3A). PAGBC significantly increased PAGBC in AMSCs at NP (Figure 3A). Interestingly, the increases in PAGBC in these conditions resulted in decreases in miR-133b, and vice versa (Figure 3B). Moreover, HP-induced PAGBC promoted osteogenic differentiation, shown by representative images (Figure 3C) and by quantification (Figure 3D), which was supported by upregulation of osteogenesis-associated genes, collagen I (Col I, Figure 3E) and osteopontin (OPN, Figure 3E). PAGBC depletion significantly attenuated the HP-induced osteogenic differentiation, shown by representative images (Figure 3C) and by quantification (Figure 3D), which was supported by attenuated increases in osteogenesis-associated genes, Col I and OPN (Figure 3E). On the other hand, overexpression of PAGBC at NP was sufficient to induce osteogenic differentiation, shown by representative images (Figure 3C) and by quantification (Figure 3D), which was supported by significant upregulation of osteogenesis-associated genes, Col I and OPN (Figure 3E). Together, these data suggest that HP induces osteogenic differentiation of AMSCs through PAGBC.

### MiR-133b inhibits RUNX2 in AMSCs

**Figure 4:**
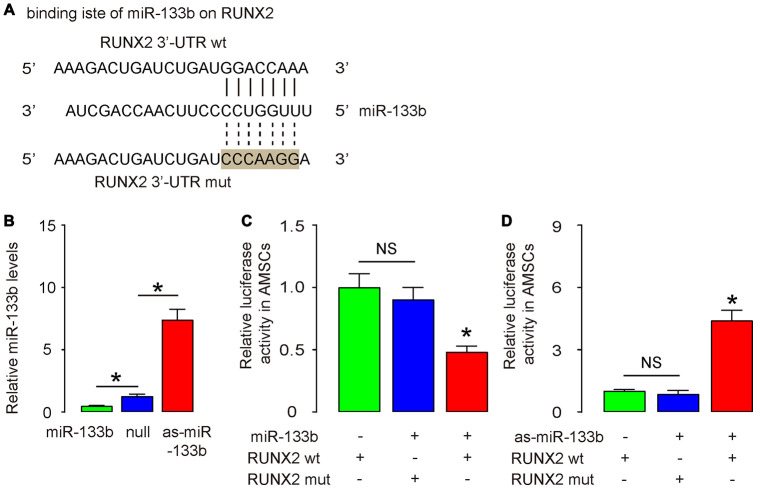
**MiR-133b inhibits RUNX2 in AMSCs.** (**A**) Bioinformatic prediction of the binding site for miR-133b on the 3’-UTR of RUNX2, which was used for generating 3'-UTR of wildtype RUNX2 mRNA (RUNX2 wt) and 3'-UTR of RUNX2 mRNA with a mutant at miR-133b-binding site (RUNX2 mut). (**B**) Plasmids that overexpress miR-133b or deplete it were prepared. Plasmids carrying a null sequence were used as controls. These plasmids were used to transfect AMSCs and RT-qPCR for miR-133b was assessed in these cells. (**C**, **D**) We prepared luciferase reporter for wildtype (wt) RUNX2 and RUNX2 with a mutant at the miR-133b binding site (mut). The luciferase reporter assay was performed on AMSCs, using either miR-133b (**C**) or as-miR-133b (**D**). N=5. *p<0.05. NS: non-significant.

RUNX2 is a key inducer for osteogenesis and has been shown to be a miR-133b-targeting gene [19]. Using bioinformatics tools, we detected the binding site for miR-133b on the 3’-UTR of RUNX2, which was used for generating 3'-UTR of wildtype RUNX2 mRNA (RUNX2 wt) and 3'-UTR of RUNX2 mRNA with a mutant at miR-133b-binding site (RUNX2 mut) (Figure 4A). Next, we examined whether the bindings of miR-133b to 3’-UTR of RUNX2 mRNA may affect protein translation of RUNX2. First, we transfected mouse AMSCs cells with plasmids carrying miR-133b or as-miR-133b or scr as a control. RT-qPCR for miR-133b was performed in transfected AMSCs to confirm the alteration of miR-133b levels (Figure 4B). Next, the RUNX2 wt and RUNX2 mut were respectively cloned into luciferase reporter plasmids. AMSCs were then co-transfected with miR-133b plasmids and one plasmid from either RUNX2 wt or RUNX2 mut, and subsequently subjected to a dual luciferase reporter assay. We found that overexpression of miR-133b reduced luciferase activity of RUNX2 wt but had no effects on RUNX2 mut (Figure 4C). In another experiment, AMSCs were then co-transfected with as-miR-133b plasmids and one plasmid from either RUNX2 wt or RUNX2 mut, and subsequently subjected to a dual luciferase reporter assay. We found that depletion of miR-133b increased luciferase activity of RUNX2 wt but had no effects on RUNX2 mut (Figure 4D). These results suggest that miR-133b specifically targets 3’-UTR of RUNX2 mRNA to inhibit its translation in AMSCs.

### miR-133b suppresses osteogenic differentiation of AMSCs through RUNX2

**Figure 5:**
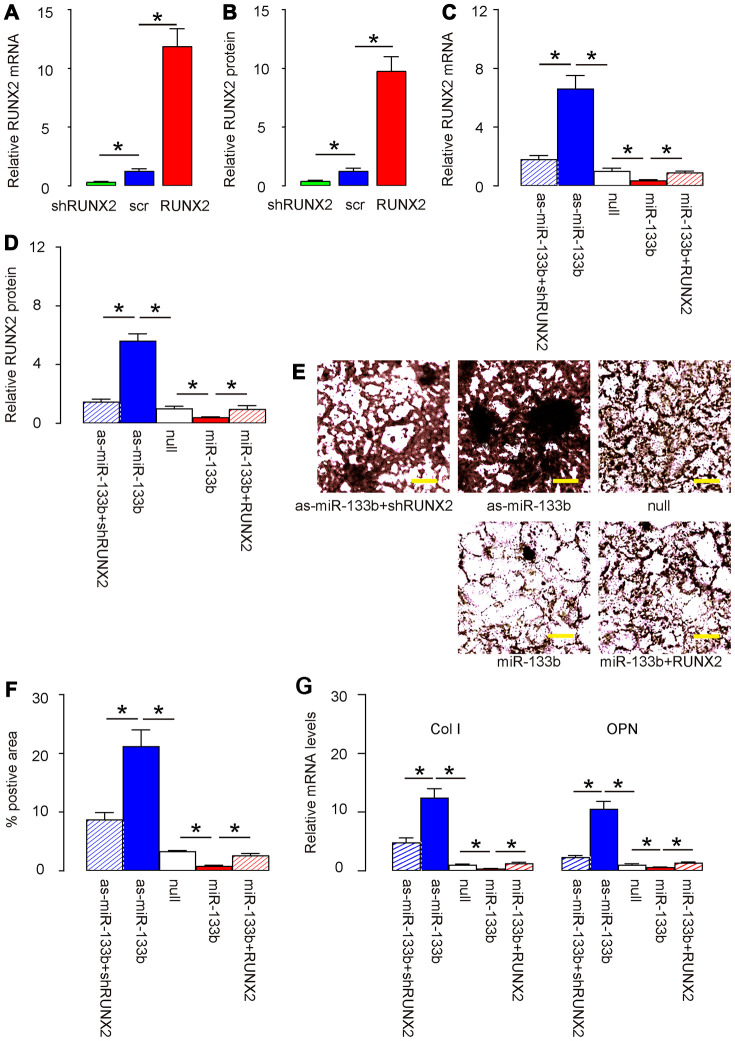
**miR-133b suppresses osteogenic differentiation of AMSCs through RUNX2.** (**A**, **B**) Plasmids that overexpress or deplete RUNX2 were validated by RT-qPCR (A) and by ELISA (**B**) for RUNX2. (**C**–**G**) AMSCs were transfected with either miR-133b or as-miR-133b, compared to null controls. In another two groups, AMSCs were co-transfected with miR-133b and RUNX2 or with as-miR-133b and shRUNX2. (**C**, **D**) RT-qPCR (**C**) and ELISA (**D**) for RUNX2. (**E**, **F**) Osteogenic differentiation of AMSCs was determined by Von kossa staining, shown by representative images (**E**) and by quantification (**F**). (**G**) RT-qPCR for osteogenesis-associated genes, collagen I (Col I) and osteopontin (OPN). N=5. *p<0.05. Scale bars are 50μm.

**Figure 6:**
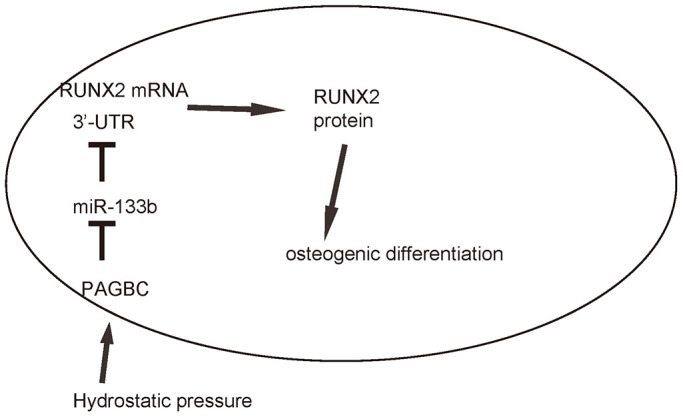
**Schematic of the model.** HP induces upregulation of PAGBC, which binds to miR-133b to prevent its suppression on translation of RUNX2, resulting in enhanced RUNX2-mediated osteogenic differentiation of AMSCs.

Finally, we assessed whether HP/PAGBC-altered miR-133b levels may regulate osteogenic differentiation of AMSCs through RUNX2. First, we prepared plasmids that overexpress or deplete RUNX2, and validated them by RT-qPCR (Figure 5A) and by ELISA (Figure 5B) for RUNX2. Next, AMSCs were transfected with either miR-133b or as-miR-133b, compared to null controls. In another two groups, AMSCs were co-transfected with miR-133b and RUNX2 or with as-miR-133b and shRUNX2. We found that as-miR-133b induced significantly increases in RUNX2 mRNA (Figure 5C) and protein (Figure 5D) in AMSCs, which were significantly attenuated by shRUNX2 (Figure 5C, 5D). On the other hand, miR-133b induced significantly decreases in RUNX2 mRNA (Figure 5C) and protein (Figure 5D) in AMSCs, which were significantly attenuated by RUNX2 (Figure 5C-D). Moreover, as-miR-133b-induced increases in RUNX2 in AMSCs resulted in increased osteogenic differentiation, shown by representative images (Figure 5E) and by quantification (Figure 5F), which was supported by significant upregulation of osteogenesis-associated genes, Col I and OPN (Figure 5G). On the other hand, miR-133b-induced decreases in RUNX2 in AMSCs resulted in decreased osteogenic differentiation, shown by representative images (Figure 5E) and by quantification (Figure 5F), which was supported by significant downregulation of osteogenesis-associated genes, Col I and OPN (Figure 5G). Thus, miR-133b suppresses osteogenic differentiation of AMSCs through RUNX2. The finding in the current study was then summarized in a schematic (Figure 6).

## DISCUSSION

Effective and optimal bone regeneration can benefit millions of patients worldwide each year. AMSCs have the advantage of being abundant, accessible and functional [3–5]. Valerie et al. recently showed that AMSCs rendered collagen deposition, hyaluronic acid, elastin levels, and the organization of elastic fibers back to normal in a chronic vocal fold scar rabbit model [8]. It was proposed that AMSCs may produce and secrete some trophic factors to enhance tissue regeneration, and may release some other factors to antagonize the pro-fibrotic factors at the site of bone regeneration. Moreover, AMSCs may adjust inflammation at site and inhibit fibrotic tissue formation [6–9]. After all, differentiation of AMSCs themselves into osteocytes contributes significantly to the bone regeneration. Nevertheless, the cellular and molecular mechanisms underlying HP-induced osteogenic differentiation of AMSCs remain poorly understood.

Reports on the role of lncRNAs in the regulation of osteogenic differentiation of AMSCs have been accumulating in recent years [26]. Specifically, next-generation sequencing to MSC transcriptomes has revealed that lncRNAs are closely associated with the osteogenic differentiation of MSCs [27]. However, most current studies have used bone marrow derived MSCs, rather than AMSCs. Moreover, none of previous studies have demonstrated the regulatory axis “PAGBC/miR-133b/RUNX2” in osteogenic differentiation of MSCs.

The focus on study of PAGBC/miR-133b/RUNX2 regulatory axis stemmed from several important findings. First, PAGBC was one of the rare lncRNAs that were induced to significantly upregulate by HP. Second, the interaction between PAGBC and miR-133b has been nicely demonstrated in tumorigenesis of gallbladder [28], but never shown in the process of osteogenic differentiation of AMSCs. The role of miR-133b in the osteogenic differentiation of AMSCs was, however, well developed [19], including its direct targeting of RUNX2 [19, 20]. Thus, our study was not only based on strong previous studies, but also conveyed significant novel information. Here, by a set of gain-of-function and loss-of-function experiments, we showed that HP induced upregulation of PAGBC, which bound to miR-133b to prevent its suppression on translation of RUNX2, resulting in enhanced RUNX2-mediated osteogenic differentiation of AMSCs. Interestingly, miR-133b seemed to reduce not only the levels of RUNX2 protein, but also the mRNA levels of RUNX2, suggesting that the binding of miR-133b to 3’-UTR of RUNX2 mRNA may result in the mRNA degradation. The only remaining question is how HP increases PAGBC expression. Although a possible explanation has been acknowledged in another report [29], the exact mechanisms remain elusive. Epigenetic modification by HP should play a center role [30–32], and could be examined in future studies.

To summarize, here we provided evidence to demonstrate a novel pathway that controls osteogenic differentiation of AMSCs through HP/ PAGBC/miR-133b/RUNX2, which should be informative for generating novel strategies for facilitating bone regeneration.

## MATERIALS AND METHODS

### Protocol approval

All experimental procedures were performed in accordance with the guidance for the Care and Use of Laboratory Animals, which was proved by the research committee of Second Military Medical University.

### HP culture and osteogenic differentiation of AMSCs

Human adipose tissue was obtained from a 35-year-old male healthy donor. After careful rinsing, the isolated adipose tissue was cut into small pieces of 1mm in diameter, and then incubated with 0.2 % collagenase I (Sigma-Aldrich, St. Louis, MO, USA) in a rotator at 37^0^C for 45 minutes for digestion. Afterwards, the collagenase I was neutralized with Dulbecco’s modified Eagle’s medium (DMEM; Gibco, San Diego, CA, USA) suppled with 10 % fetal bovine serum (FBS; Gibco) to stop digestion and then the suspension was filtered through a 200 mm nylon mesh to discard the undigested tissue. The obtained cells were re-suspended in DMEM supplemented with 15 % FBS for 10 passages’ positive selection of the attached cells. A positive clone was then subjected to flow cytometry analysis to determine the MSC phenotype. For osteogenic induction, cultured AMSCs were subjected to Osteogenic Differentiation Toolkit [American Type Culture Collection (ATCC), Rockville, MD, USA; Catalog number: PCS-500-052]. Von kossa staining was used to detect osteocyte differentiation after 10 days’ culture in the differentiation media. The quantification of the positive area was done using NIH Image J software (Bethesda, MA, USA). For long-term cell culture under high HP, we used an apparatus, in which cells were cultivated in the flowing medium pumped constantly from medium reservoir equilibrated with adequate concentrations of CO_2_ and O_2_. The HP in the chamber was created by setting a resistant backpressure regulator in the runoff road of the culture medium. The normal pressure was set as 0 psi, while HP was set as 100 psi.

### Transfection of AMSCs

Transfection of AMSCs was done using several sets of plasmids with Lipofectamine 3000 reagent (Invitrogen, St. Louis, MO, USA). Set 1: plasmids carrying miR-133b, or antisense for miR-133b (as-miR-449), or a null sequence (null) as a control. Set 2: plasmids carrying PAGBC or shRNA for PAGBC (shPAGBC), or a scramble sequence (scr) as a control. Set 3: plasmids carrying RUNX2 or shRNA for RUNX2 (shRUNX2), or a scramble sequence (scr) as a control. Full length of PAGBC was obtained by PCR using 5’-RACE and 3’-RACE analyses with GeneRacerTM kit (Invitrogen) according to the manufacturer's instructions. Primers are 5’-CTACTCTTTACCTCCTCCCAACCATT-3’, 5’-CCCAGTTCCTTAGAATCTTCAGTTGC-3’, 5’-AGAAAGTTGGAGCAAAGGTTTGGCC-3’ and 5’-CCTCTTGCAGGAAGGATGGATTCTC-3’. The sequence for shPAGBC that targeted the sequence of PAGBC at the miR-133b binding site was 5’-CGGGTGTCTTTTGCTCTGCAGTCA-3’. The shRUNX2 sequence was 5’-CAGACAAGUGAAGAGGUUUU-3’. The complete coding sequence for RUNX2 was prepared by PCR using human osteocyte cDNA as a template. All plasmids carried a green fluorescent protein (GFP) reporter to allow quantification of transfection efficiency.

### Flow cytometry

For flow cytometric analysis, antibodies were FITC-conjugated anti-CD73, CD90, CD105, CD34, CD45 and HLA-DR (Becton-Dickinson Biosciences, San Jose, CA, USA). Data were analyzed using FlowJo software (Flowjo LLC, Ashland, OR, USA).

### ELISA

Proteins were isolated from cultured cells. ELISA for RUNX2 was performed using an anti-human RUNX2 ELISA kit (LS-F4390, LSBio, Seattle, WA, USA).

### Quantitative PCR (RT-qPCR)

Total RNA was extracted from cells using miRNeasy mini kit (Qiagen, Hilden, Germany) for cDNA synthesis. RT-qPCR was performed in duplicates with QuantiTect SYBR Green PCR Kit (Qiagen). All primers were purchased from Qiagen. Data were collected and analyzed using 2-ΔΔCt method. Values of genes were first normalized against GAPDH, and then compared to experimental controls.

### Bioinformatics and proven experiments

The interaction between miRNAs and 3’-UTR of mRNAs was determined by TargetScan, using the context++ score system, as described [33]. The dual-luciferase reporter plasmids, p3’-UTR-RUNX2 (containing the wild-type RUNX2 3’-UTR binding site in luciferase reporter plasmid and p3’-UTR-RUNX2-mut (containing the mutant RUNX2 3’-UTR; mut) were constructed in RiboBio Co. Ltd (Shanghai, China). For the luciferase assay, the constructed 3’-UTR plasmid and miR-133b/as-miR-133b were co-transfected into AMSCs using LipofectamineTM 3000. The luciferase activity was detected with the dual-luciferase reporter assay system (Promega, Shanghai, China) after co-transfection of the cells for 48 hours, following the manufacturer’s protocol. The binding site between PAGBC and miR-133b was predicted by miRcode (http://www.mircode.org/mircode) and StarBase (http://starbase.sysu.edu.cn/). The fragments of PAGBC containing the predicted wild-type (wt) and mutant (mut) miR-133b-binding sites were cloned into pmirGLO reporter vectors (Promega Corporation, Madison, WI, USA) to generate the PAGBC-wt and PAGBC-mut plasmids, respectively.

### Statistical analysis

GraphPad prism software (GraphPad Software, Inc. La Jolla, CA, USA) was used for statistical analyses. Unpaired two-tailed Student t test was applied for comparison between two groups. One-way ANOVA with a Bonferroni correction was applied for comparison among several groups. Data were represented as mean ± SD and were considered significant if p<0.05.
